# Domestic Service in Military Hospitals

**Published:** 1918-06-08

**Authors:** 


*To the Editor of* The Hospital.

### Domestic Service in Military Hospitals.

*To the Editor of* The Hospital.

Sir,—Do you not think it is time that the War Office recognised domestics in all military hospitals as war workers and allowed those over eighteen years of age the privilege of wearing a uniform such as that worn by the general service V.A.D., and those maids under eighteen years of age a medal? Writing from my own experience, I have now worked hard and done my bit to the very best of my ability for nearly three and a half years in the same military hospital, and have nothing to prove that I am a war worker, and am very probably looked upon by some as a slacker. Of course, we domestics quite realise that to a certain extent we have the remedy in our own hands, and could give up our situations and join the V.A.D., but do you not think that may do more harm than good? Why should all experienced maids give up when a little consideration on the part of those in authority could soon put matters right? Most of us would be quite willing to buy our own uniform, agree to any rules made, go on doing our bit, and sign on for the duration of the war, while we did our best to do credit to our uniforms. Unseen War-worker.

[This letter is dealt with in "Round the Hospitals" this week, p. 212.—Ed. The Hospital.]

